# Anisohydric sugar beet rapidly responds to light to optimize leaf water use efficiency utilizing numerous small stomata

**DOI:** 10.1093/aobpla/plaa067

**Published:** 2020-12-02

**Authors:** Georgina E Barratt, Debbie L Sparkes, Lorna McAusland, Erik H Murchie

**Affiliations:** School of Biosciences, University of Nottingham, Loughborough, UK

**Keywords:** Anisohydric, speed of stomatal response, stomatal density, stomatal size, sugar beet, water use efficiency, wilting

## Abstract

Under conditions of high transpiration and low soil water availability, the demand for water can exceed supply causing a reduction in water potential and a loss of cell turgor (wilting). Regulation of stomatal aperture mediates the loss of water vapour (*g*_s_), which in turn is dependent in part on the anatomical characteristics of stomatal density (SD) and stomatal size (SS). Anisohydric sugar beet (*Beta vulgaris*) is atypical, exhibiting wilting under high soil water availability. Spinach (*Spinacia oleracea*) belongs to the same family *Chenopodiaceae s.s*., but demonstrates a more typical wilting response. To investigate the role of stomatal dynamics in such behaviours, sugar beet and spinach leaves were exposed to step-changes in photosynthetic photon flux density (PPFD) from 250 to 2500 µmol m^−2^ s^−1^. Using a four log-logistic function, the maximum rate of stomatal opening was estimated. Concurrent measurements of SD and SS were taken for both species. While sugar beet coupled faster opening with smaller, more numerous stomata, spinach showed the converse. After exposure to drought, maximum *g*_s_ was reduced in sugar beet but still achieved a similar speed of opening. It is concluded that sugar beet stomata respond rapidly to changes in PPFD with a high rate and magnitude of opening under both non-droughted and droughted conditions. Such a response may contribute to wilting, even under high soil water availability, but enables photosynthesis to be better coupled with increasing PPFD.

## Teaser

Sugar beet is an important crop that is surprisingly prone to short-term reversible wilting of leaves. This wilting is driven by pores on the leaf surface (stomata) remaining open for photosynthesis when they would normally close in response to lowered water potential. This is termed anisohydric behaviour. The benefits to the plant are still unclear but here we show that sugar beet have an adaptation which helps improve function under these conditions. They possess many smaller stomata which rapidly respond to changes in light intensity, improving leaf productivity but also causing the high leaf evaporation which is associated with wilting.

## Introduction

The largest areas of sugar beet (*Beta vulgaris* ssp. *vulgaris*) production are in Europe, Russia and North America, where it is grown for both sugar production and biofuel (Draycott 2006). Its wild ancestor is sea beet (*Beta vulgaris* ssp. *maritima*), which is thought to be the origin of the crop’s salinity tolerance and suitability for the temperate climates in which sugar beet is grown (Ribeiro *et al.* 2016). Although sugar beet yields are increasing in the UK, losses of up to 25 % are evidenced in the driest years (Jaggard *et al.* 1998). Improving the resilience of the crop is important to maintain yields into the future as the world’s climate changes and hotter, drier summers are predicted in the UK (David 2017). A number of studies have shown that drought tolerance varies between sugar beet genotypes and is associated with a range of traits from specific leaf weight to maintenance of canopy green area (Pidgeon and Jaggard 1998; Ober *et al*. 2004, 2005; Rajabi *et al.* 2009) but these studies did not assess how sugar beet regulate water use efficiency at the leaf level. Regulation of stomatal aperture mediates the rate of stomatal conductance (*g*_s_) and assimilation (*A*) and it is the ratio of these two processes which gives a value for intrinsic water use efficiency (WUE_i_); hence, the anatomical characteristics of stomatal density (SD) and stomatal size (SS) are important in determining these processes. Therefore, to understand WUE_i_ in sugar beet, SD and SS and the effect these parameters have on the magnitude and speed of stomatal response must be understood.

A distinctive trait of the sugar beet crop is its tendency to wilt on bright and warm days, even when water is available in the soil profile. Research by Kohl and Cary (1969) demonstrated that light mist irrigation can reduce the prevalence of wilting. This suggests stomata are not closing as leaf water potential (Ψ _L_) falls and that high levels of transpiration drive the wilting response. The reluctance of sugar beet stomata to close is attributed to reduced stomatal sensitivity to falling Ψ _L_ and high levels of osmotic adjustment, rather than stomatal closure to reduce water losses through transpiration, which results in a rapid decline in Ψ _L_ over the day (McCree and Richardson 1987). Plants that do not maintain a stable midday Ψ _L_, including sugar beet, are described as an anisohydric, as opposed to isohydric plants which maintain midday Ψ _L_ (Tardieu and Simonneau 1998). Despite wilting, the anisohydric response enables high photosynthetic rates to be maintained for longer periods than in isohydric plants, which close stomata sooner, and are suited to environments where water is abundant and droughts are short and of moderate severity (Sade *et al.* 2012). Key to the observation that sugar beet is anisohydric is the relationship between stomata and the environment and exploring this could identify if stomatal responses are a driver of wilting under high soil water availability.

Stomata respond to signals derived from the external and internal leaf environment to reduce water loss through transpiration and maximize CO_2_ assimilation (Lawson *et al.* 2010). Declining plant water status (affected by factors such as vapour pressure deficit (VPD) (Nonami *et al.* 1991), soil water potential (Zhang and Davies 1990) and Ψ _L_ (Brodribb and Holbrook 2003)), rising CO_2_ concentrations in the intercellular air spaces (Xu *et al*. 2016) and low PPFD promote stomatal closure (Shimazaki *et al*. 2007), whilst the opposite conditions drive opening. For optimal WUE_i_ stomata should open quickly in response to favourable conditions, to a magnitude which supports maximum *A*, without overshooting which would result in excessive *g*_s_ and water loss (McAusland *et al.* 2016). There are a range of approaches to assess the impact of changing environmental variables on the speed and magnitude of stomatal response and most studies develop a model based on the sigmoidal response to step-changes in light (Kirschbaum *et al.* 1988; Assmann and Grantz 1990; Knapp 1993; Zipperlen and Press 1997; Vico *et al.* 2011; Drake *et al.* 2013). Step-changes in light are more representative of the field environment and facilitate plant responses more representative of those in the field compared to light curves in which light intensity changes gradually. This approach identifies the maximum and minimum rates of *g*_s_ (*g*_smax_, *g*_smin_) and *A* (*A*_max_, *A*_min_) and the rate of change between the minimum and maximum giving a value for the speed of stomatal response in dynamic light (Kirschbaum *et al.* 1988). A popular approach is that of Knapp (1993) which uses a time constant to identify where 63 % of the magnitude of the change has occurred to give a measurement of the time taken to reach this point, whilst other studies derive values from different points such as 50 % (Drake *et al.* 2013) and 90 % of the maximum value for *g*_s_ or *A* (Zipperlen and Press 1997). Alternatively, the change in stomatal response divided by the change in time between 10 and 90 % of the magnitude of the light pulse can be used as a more simplistic approach (Assmann and Grantz 1990). The model chosen depends on the hypothesis to be addressed and can be dependent on the asymmetry of opening and closing, which can be species- and environment-dependent (Vico *et al.* 2011).

The speed of stomatal response to dynamic conditions has a significant influence on WUE_i_ and is related to the plant’s SD and SS (Drake *et al.* 2013; Lawson and Vialet-Chabrand 2019), which have an inverse relationship in most species (Franks *et al.* 2009). A greater SD and reduced SS is typically associated with faster stomatal responses which increases the coordination between *A* and *g*_s_ and increases WUE_i_ (Lawson and Weyers 1999; Lawson *et al.* 2010; McAusland *et al.* 2016; Vialet-Chabrand *et al.* 2017), although this may not improve WUE_i_ over a longer time scale (Moualeu-Ngangue *et al.* 2016). Given the different factors influencing stomatal dynamics, it is important to assess species individually and to understand the relationship between SD and SS, how this affects the speed of stomatal response and the impact this has on *g*_s_ and *A*, and consequently WUE_i_.

This study used dynamic light to assess the magnitude and speed of stomatal response and the relationship with SD and SS to enable an assessment of *g*_s_, *A* and WUE_i_ and identify if stomatal responses could be a driver of wilting in sugar beet. The hypothesis was that slow stomatal closure in sugar beet is attributed to a low SD and large SS which leads to a disconnect between *g*_s_ and *A* and excessive water loss from transpiration. To address this hypothesis, spinach was selected as a comparison species as it also belongs to the family *Chenopodiaceae s.s.* but demonstrates a more typical wilting response. In addition to this it was hypothesized that water stress and wilting, which is often evident in the sugar beet crop, would alter the speed of stomatal response compared to well-watered plants to conserve water and increase WUE_i_ at the expense of carbon fixation.

## Materials and Methods

### Plant material

Sugar beet (*Beta vulgaris* ssp. *vulgaris*) cv. Haydn and spinach (*Spinacia oleracea*) cv. Mikado were sown in 5-L pots containing a 1:1 mix of Kettering loam and sand and grown in a controlled environment room. Pots were placed on raised benches in a randomized block design, with eight replicates of each species, under fluorescent tubes (LUMILUX HO 54W/840 T5, Osram, Munich, Germany) which provided 12 h of light followed by 12 h of darkness, with an hour dawn and evening light adjustment. Three seeds were sown per pot and thinned to a single plant at 40 days after sowing (DAS) and hand-watered to prevent soil drying. Humidity was between 44 and 85 % with a daytime temperature of 22 ± 3 °C and night-time temperature of 6 ± 1 °C, monitored using a humidity and temperature data logger (TinyTag Ultra 2, Gemini Instruments, Chichester, UK). A split application totalling 1.05 g of ammonium nitrate was applied in solution with 15 mL applied at 35 DAS and 39 DAS.

### Drought treatment

Water was withdrawn from blocks 1 and 2 at 119 DAS and blocks 3 and 4 at 121 DAS for the drought treatment. The staggered water withdrawal ensured that the water deficits were comparable when measurements were taken, as each block took a day to measure. A capacitance soil moisture probe (ML 3 ThetaProbe, Delta T, Cambridge, UK) was used to monitor soil moisture content. The probe was inserted into the soil to 5 cm and percentage soil moisture recorded for each plant as gas exchange measurements were being taken. The spinach did not reach a water-stressed state as there was no wilting or decline in *A*_max_ in the time constraints of the experiment whilst wilting was evident in the sugar beet. The drought responses are therefore focused on the results from the sugar beet observations.

### Gas exchange and chlorophyll fluorescence measurements

Leaves were dark-adapted for 30 min by wrapping in aluminium foil. The room was fully darkened when the leaves were unwrapped and placed into infrared gas analyser cuvette (LI-6800, LI-COR, Lincoln, NE, USA) with help of a green LED head torch (LUMii 10-465-200, LUMii, Coventry, UK) providing minimal light for the operator. Leaf 7–8 and 9–10 were used for the non-droughted and droughted measurements, respectively, and selected to ensure a uniform size, with spinach leaves of the same age as the beet leaves being selected for measurement.

Gas exchange measurements were taken using infrared gas analyser (LI-6800, LI-COR, Lincoln, NE, USA). An auto log program within a control loop set PPFD in the gas exchange cuvette at 250 µmol m^−2^ s^−1^ for 15 min, 2500 µmol m^−2^ s^−1^ for 30 min and 250 µmol m^−2^ s^−1^ for 30 min. The maximum light intensity was identified following standard light–response curve procedures with 200 µmol m^−2^ s^−1^ PPFD step-increases in light intensity every 5 min and identifying the level at which *A* plateaued in both beet and spinach. The minimum light intensity was chosen as 10 % of this maximum light intensity. Gas exchange measurements of *g*_s_, *A*, and leaf VPD and chlorophyll fluorescence parameters of *F*_v_′/*F*_m_′ (maximum photosystem II (PSII) efficiency in the light), ΦPSII (quantum efficiency of PSII electron transport in the light) and *q*_p_ (photochemical quenching) were logged every minute of the 75-min program (15 at low light T1–T15, 30 at high light T16–T45 and a further 30 at low light T46–T75) using a multiphase flash fluorometer (LI-6800 multiphase flash fluorometer, LI-COR, Lincoln, NE, USA) (flash was 300 ms and 10 000 µmol m^−2^ s^−1^).

Standard settings were; flow 500 μmol s^−1^, reference CO_2_ 400 μmol, RH 50 % and leaf temperature 20 ± 3 °C, with matching at every measurement. The sugar beet and spinach measurements were taken at 90, 91, 92 and 96 DAS on blocks 1, 2, 3 and 4, respectively. The sugar beet non-droughted and droughted measurements were taken at 124, 125, 126 and 127 DAS on blocks 1, 2, 3 and 4, respectively. The VPD maintained in the LI-6800 chamber was between 1 and 1.2 KPa for the both the beet and the spinach **[see****Supporting Information—Fig. S1A****]**, and for the non-droughted and droughted beet at the low light levels **[see****Supporting Information—Fig. S1B****]**. The spike at the onset of high light is due to the LI-6800 adjusting to maintain cuvette temperature and RH % as the stomata open and transpire. Once settled at high light VPD significantly (*P* < 0.001) increased to between 1.3 to 1.4 KPa for the beet and the spinach and between 1.1 to 1.3 KPa (*P* = 0.009) for the non-droughted and droughted beet. There was no significant difference in VPD between the sugar beet and spinach and the non-droughted and droughted beet.

### Modelling the light response

**Figure 1.**
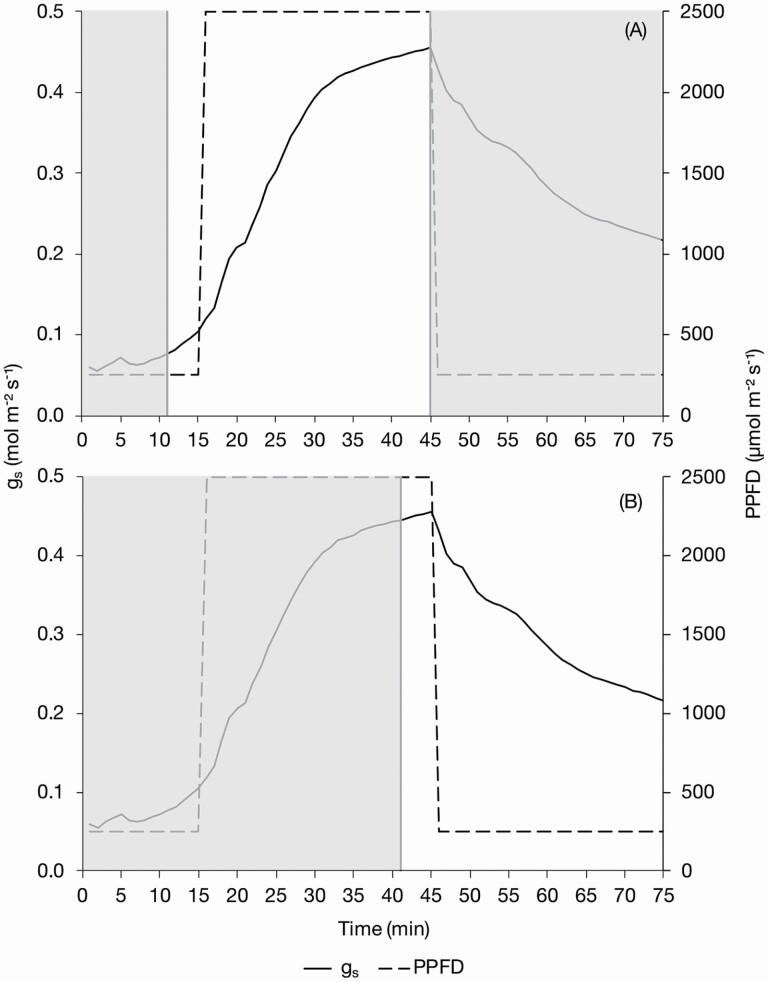
Stomatal conductance measured over a 75-min program (T1–T75) which was used to model stomatal opening and closing. Plants were exposed to 250 µmol m^−2^ s^−1^ for 15 min (T1–T15) followed by 2500 µmol m^−2^ s^−1^ for 30 min (T16–T45) and 250 µmol m^−2^ s^−1^ for another 30 min (T46–T75). To model stomatal opening an LL.4 function was used with the stomatal opening curve fitted using points T11–T45 (A) and the closing phase T41–T75 (B), which are located in the non-shaded regions of the figures.

For the analysis of the speed of stomatal response, dose–response curves (DRCs) were calculated for each replicate using the *g*_s_ data in the statistical programming and graphics package R (R Core Team 2019) using the freely accessible DRC package (Ritz *et al.* 2015). Model selection by comparison of different functions was utilized to identify which log-logistic function was most suited to the data set with log logistics 4 (LL.4) producing the best fit. Log-logistic curves require a stable start and end point to enable a realistic estimate of the upper and lower limit. For this reason, the 75 data points were split into a stomatal opening (switch from 250 to 2500 µmol m^−2^ s^−1^ PPFD) and a stomatal closing (switch from 2500 to 250 µmol m^−2^ s^−1^ PPFD) phase with 35 data points in each. The opening phase consisted of points T11–T45 (i.e. 11–45 min) (Fig. 1A), as *g*_s_ was not consistently stable at T1–T10, and the closing phase T41–T75 (Fig. 1B). For opening, the first five data points (T11–T15) were therefore at low light to provide an estimate of the lower limit. The remaining 30 data points were then at high light (T16–T45) with stomatal conductance starting to plateau by the end of this period for estimation of the upper limit. For closing, the last 5 min of the high light period was used (T41–T45) to establish an upper limit followed by the 30 min of low light (T46–T75), with conductance starting to plateau at the end of this period for estimation of the lower limit. The estimated lower (OE*g*_smin_—at opening, CE*g*_smin_—at closing) and upper (OE*g*_smax_—at opening, CE*g*_smax_—at closing) limit to stomatal conductance (*g*_s_) calculated using the LL.4 curve, could then be compared to the measured lower (*g*_smin_) and upper (*g*_smax_) *g*_s_ values from the LI-COR. The point halfway between the estimated lower and upper limits of stomatal conductance (Og_s_50—at opening, Cg_s_50—at closing) and the slope of the tangent of the line at the Og_s_50 or Cg_s_50 provides an estimate of the speed of stomatal closure at that point for opening or closing, respectively. The mean curve parameters for the treatments were calculated using the LL.4 curves from each replicate and the mean LL.4 curves for each treatment compared using two-way ANOVA in R to identify if treatments produced significantly different curves.

### Calculating intrinsic water use efficiency

Intrinsic water use efficiency was calculated using Equation (1) (Condon *et al.* 2002). The values for *A* and *g*_s_ were collected using the infrared gas analyser as previously outlined.


$$\begin{array}{l} WUE_{i}=\;\frac{A}{g_{s}} \end{array}$$ (1)

### Stomatal anatomy

A stomatal impression of the abaxial and adaxial leaf surface of the gas exchange measurement leaf of each sugar beet and spinach replicate was taken after the non-droughted measurements at 97 DAS. Clear nail varnish was applied and left to dry for 20 min until no longer tacky. Clear tape was applied to the area and peeled to lift the dried varnish which was mounted on a microscope sample slide. Three images were taken from each sample slide using a microscope (Leica 5000B, Leica, Wetzlar, Germany) with a light source (Leica CTR5000, Leica, Wetzlar, Germany) at 100× magnification and cropped to 1 mm^2^ using the microscope scale for reference in Fiji (Schindelin *et al.* 2012). The stomata in the cropped images were manually counted using the Cell Counter plugin, with an average SD value of the abaxial and adaxial leaf surface calculated for each replicate from the three 1 mm^2^ areas counted.

Stomatal size was calculated by reducing the 1 mm^2^ image to 0.25 mm^2^ and randomly selecting 10 stomata to be measured. The stomatal pore (SP) length, peristomatal groove (PSG) length and guard cell (GC) width were measured and maximum theoretical conductance calculated for the adaxial and abaxial leaf surface using the method of Franks *et al.* (2009).

### Statistical analysis

Repeated-measures ANOVA was performed on the *g*_s_, *A*, *F*_v_′/*F*_m_′, ΦPSII, *q*_p_ and WUE_i_ data with time as the independent variable and a two-way ANOVA on the stomatal impressions data sets with species as the independent variable. Anomalous WUE_i_ values in excess of 200 at T17 were removed from the analysis as these were caused by the LI-COR automatically adjusting to the sudden increase in *g*_s_ and *A* at the onset of high light to achieve the temperature and RH set points. GenStat 15th edition (VSN International Ltd, Hemel Hempstead, UK) was used for the statistical analyses except for the curve fitting which was performed in R as previously described.

## Results

### Sugar beet and spinach

#### Speed of response to light in beet and spinach.

**Figure 2.**
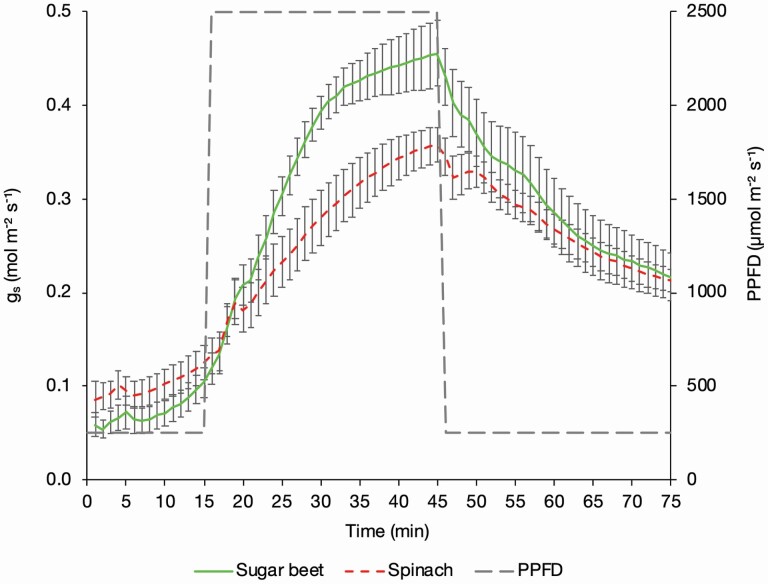
The stomatal conductance of sugar beet and spinach plants exposed to changing PPFD. Plants were exposed to a PPFD of 250 µmol m^−2^ s^−1^ for 15 min, 2500 µmol m^−2^ s^−1^ for 30 min and 250 µmol m^−2^ s^−1^ for 30 min, measured using an infrared gas analyser (LI-6800, LI-COR, Lincoln, NE, USA) with measurements logged every minute. These data were used to plot LL.4 curves and estimate stomatal speed. Error bars show SE ±, *n* = 8 sugar beet and 8 spinach.

**Figure 3.**
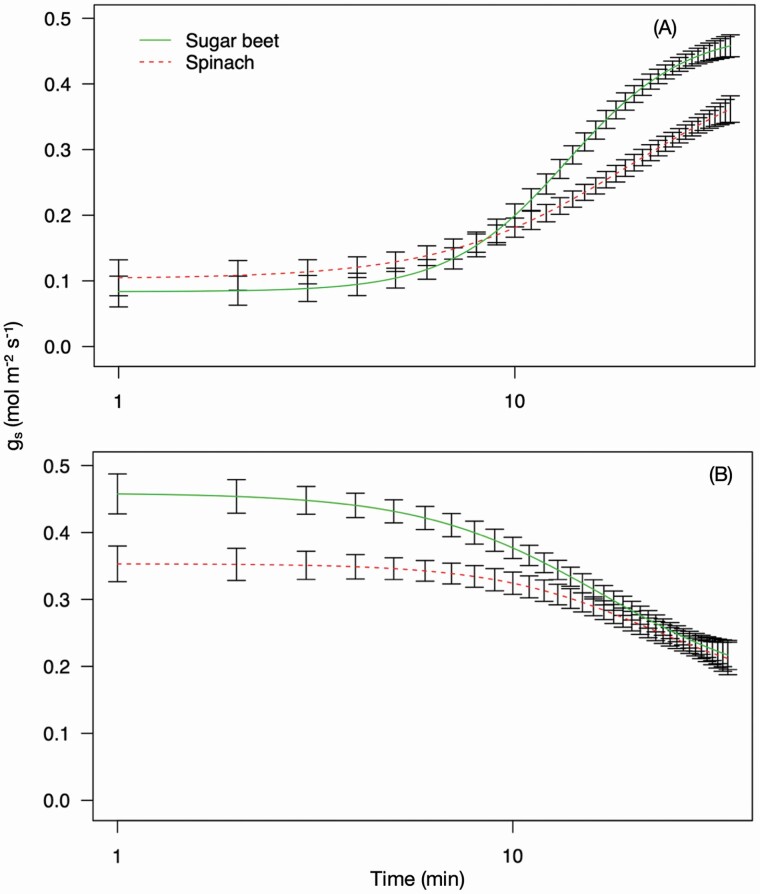
The LL.4 curves of stomatal conductance (*g*_s_) of sugar beet and spinach. Stomatal conductance was measured using an infrared gas analyser (LI-6800, LI-COR, Lincoln, NE, USA) and fitted using plotted using the DRC package (Ritz *et al.* 2015) in the statistical programming and graphics package R (R Core Team 2019). The plants were exposed to a PPFD of 250 µmol m^−2^ s^−1^ for 15 min, 2500 µmol m^−2^ s^−1^ for 30 min and 250 µmol m^−2^ s^−1^ for 30 min. Curve (A) shows the curve fitted when using the measurements taken during the last 5 min of the initial low light period and the 30-min high light period. Curve (B) shows the curve fitted when using the measurements taken during the last 5 min of the high light period and the 30 min low light period. The curves were identified as being significantly different (*P* < 0.001) using a two-way ANOVA. Error bars show SE ± *n* = 8 sugar beet and 8 spinach.

 The sugar beet and spinach responded differently to the onset of high light (stomatal opening) and subsequent low light (stomatal closing) (Fig. 2). By fitting the LL.4 model and running a two-way ANOVA the two curves were identified as being significantly different (*P* < 0.001) (Fig. 3). The stomatal opening (Fig. 3A) of the sugar beet was faster with Og_s_50 estimated to be reached at 13.56 ± 0.60 min compared with 19.62 ± 4.87 min for the spinach (Table 1). At the estimated Og_s_50 the sugar beet stomata were still continuing to open rapidly and at a greater rate than the spinach with a slope of 2.91 ± 0.40 compared to 1.84 ± 0.52 (Table 1). The rapid opening of the sugar beet stomata was associated with a higher OE*g*_smax_ of 0.48 ± 0.02 mol m^−2^ s^−1^ (Table 1) which is close to the measured *g*_smax_ of 0.46 ± 0.04 mol m^−2^ s^−1^ at T45, which is the last measurement taken during the 30-min high light period (Table 1). The OE*g*_smax_ of spinach was 0.45 ± 0.08 mol m^−2^ s^−1^ but the *g*_smax_ reached at T45 was much lower at 0.36 ± 0.02 mol m^−2^ (Table 1), indicating that the spinach stomata were still in the process of opening at the end of the 45-min high light period. Both species had similar levels of *g*_s_ prior to the onset of high light (Fig. 2) with the sugar beet OE*g*_smin_ of 0.08 ± 0.01 mol m^−2^ s^−1^ slightly less than the 0.10 ± 0.02 mol m^−2^ s^−1^ of the spinach, with both of these estimates close to the measured *g*_smin_ at T11 (Table 1).

**Table 1.** Estimated *g*_s_ parameters from LL.4 curves of sugar beet and spinach exposed to stepwise changes in light to induce stomatal opening (250 to 2500 µmol m^−2^ s^−1^ PPFD) and closing (2500 to 250 µmol m^−2^ s^−1^ PPFD), with measured *g*_smin_ and *g*_smax_ values for comparison. The average LL.4 curves of sugar beet and spinach, plotted from eight replicates each, were analysed using two-way ANOVA and shown to be significantly different (*P* < 0.001).

		Beet			Spinach		
Parameter	Units	Output	SE	*P*-value	Output	SE	*P*-value
Opening							
OE*g*_smin_	mol m^−2^ s^−1^	0.08	0.01	<0.001	0.10	0.02	<0.001
OE*g*_smax_	mol m^−2^ s^−1^	0.48	0.02	<0.001	0.45	0.08	<0.001
Og_s_50	min	13.56	0.60	<0.001	19.62	4.87	<0.001
Slope		2.91	0.40	<0.001	1.84	0.52	<0.001
T11 *g*_smin_^a^	mol m^−2^ s^−1^	0.08	0.01	–	0.11	0.02	–
T45 *g*_smax_^b^	mol m^−2^ s^−1^	0.46	0.04	–	0.36	0.02	–
Closing							
CE*g*_smin_	mol m^−2^ s^−1^	0.16	0.06	<0.001	0.16	0.10	ns
CE*g*_smax_	mol m^−2^ s^−1^	0.46	0.02	<0.01	0.35	0.01	<0.001
C*g*_s_50	min	16.81	3.69	<0.001	22.41	10.59	<0.01
Slope		1.90	0.55	<0.001	2.16	1.17	ns
T75 *g*_smin_^c^	mol m^−2^ s^−1^	0.22	0.03	–	0.21	0.01	–

^a^Measured *g*_smin_ at T11 (pre-high light).

^b^Measured *g*_smax_ at T45 (during high light).

^c^Measured *g*_smin_ at T75 (post-high light).

The stomatal closure LL.4 curves of the sugar beet and spinach (Fig. 3B) were also significantly different (*P* < 0.001). For both species, the rate of stomatal closure was slower than opening but the sugar beet was again faster than spinach, despite reaching a higher rate of *g*_s_ in the high light period, with an estimated Cg_s_50 of 16.81 ± 3.69 min and 22.41 ± 10.59 min, respectively (Table 1). At the estimated Cg_s_50 the sugar beet had a slightly slower rate of closure with a slope of 1.90 ± 0.55 compared to 2.16 ± 1.17 in the spinach (Table 1). This may be attributed to the sugar beet having an initially rapid rate of closure which enabled it to reach a similar level of *g*_s_ quickly (Fig. 3B), which had slowed by the Cg_s_50. The CE*g*_smax_ of the sugar beet and the spinach was calculated to be 0.46 ± 0.02 mol m^−2^ s^−1^ and 0.35 ± 0.01 mol m^−2^, respectively, which is similar to the measured *g*_smax_ at T45 of 0.46 ± 0.04 mol m^−2^ s^−1^ and 0.35 ± 0.02 mol m^−2^ s^−1^ (Table 1). The CE*g*_smin_ from the closing curves was the same for the beet and spinach, and close to the measured C*g*_smin_ at T75 for both species. The *g*_s_ after the high light exposure (T46–T75) was higher than the pre-high light *g*_s_ (T1–T15) (Table 1) because the plants did not return to the dark-adapted state in which they started.

#### Assimilation and WUEi in sugar beet and spinach.

**Figure 4.**
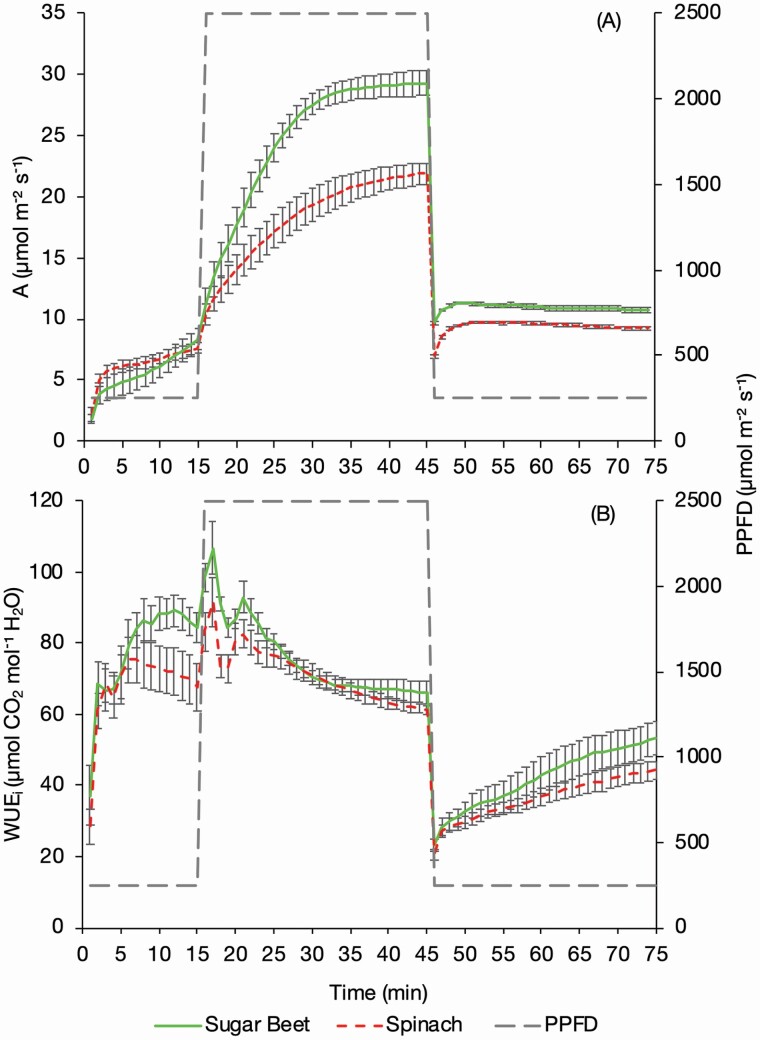
The assimilation (A) and WUE_i_ (B) of sugar beet and spinach plants exposed to changing PPFD. Plants were exposed to a PPFD of 250 µmol m^−2^ s^−1^ for 15 min, 2500 µmol m^−2^ s^−1^ for 30 min and 250 µmol m^−2^ s^−1^ for 30 min, measured using an infrared gas analyser (LI-6800, LI-COR, Lincoln, NE, USA) with measurements logged every minute. Error bars show SE ± *n* = 8 sugar beet and 8 spinach.

More open stomata facilitate greater *g*_s_ and *A*; therefore, both *g*_s_ (*P* = 0.007) (Fig. 2) and *A* (*P* < 0.001) (Fig. 4A) were significantly greater in the sugar beet than the spinach in the high light and subsequent low light period. The sugar beet reached an *A*_max_ of 29.31 ± 1.04 µmol m^−2^ s^−1^ at T45 compared to 21.87 ± 0.86 µmol m^−2^ s^−1^ in the spinach (*P* < 0.001). The sugar be*et al*so achieved significantly higher rates of *A* in the low light of 10.74 ± 0.13 µmol m^−2^ s^−1^ at T75 compared to 9.21 ± 0.22 µmol m^−2^ s^−1^ in the spinach (*P* < 0.001). These values are greater than the post-dark adaptation values at T15, the end of the initial low light period, because the plants had by then undergone high light induction.

At the onset of low light the decoupling of *g*_s_ and *A* is evident in both sugar beet and spinach as *A* declines almost instantly to a steady state due to the light requirement for photosynthesis (Fig. 4A) whilst *g*_s_ declines more slowly (Fig. 2). When averaged over the whole response curve *g*_s_ was not significantly different between the sugar beet and the spinach but *A* was (*P* = 0.002). This is evident from T9 to T15 in the initial low light phase, at the onset of high light from T16 to T23, and at the end of the second low light phase from T68 onwards (Fig. 3B). The greater ratio of *A* to *g*_s_ (i.e. WUE_i_) in the sugar beet over these time points therefore resulted in a trend of higher WUE_i_ in the sugar beet during the initial low light phase, the start of the high light phase and then again later in the second low light phase (*P* = 0.075) (Fig. 4B).

### Chlorophyll fluorescence

**Figure 5.**
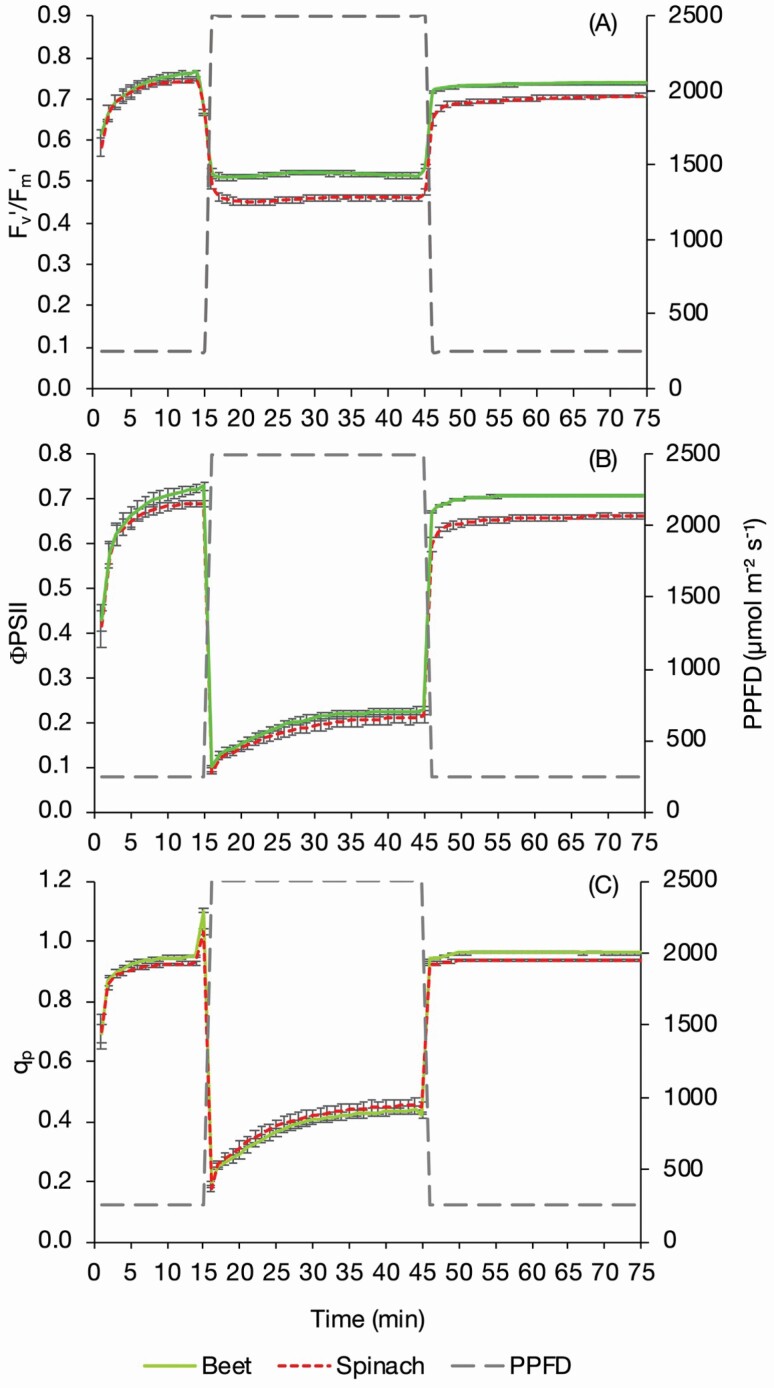
The *F*_v_′/*F*_m_′ (A), ΦPSII (B), *q*_p_ (C) and of sugar beet and spinach plants exposed to changing PPFD. Plants were exposed to a PPFD of 250 µmol m^−2^ s^−1^ for 15 min, 2500 µmol m^−2^ s^−1^ for 30 min and 250 µmol m^−2^ s^−1^ for 30 min, measured using an infrared gas analyser (LI-6800, LI-COR, Lincoln, NE, USA) with measurements logged every minute. Error bars show SE ± *n* = 8 sugar beet and 8 spinach.

Maximum PSII efficiency in the light (*F*_v_′/*F*_m_′) was not significantly different between species once the plants were stable at T10 (Fig. 5A). During the high light period differences in *F*_v_′/*F*_m_′ were evident between the beet and spinach with the beet maintaining a significantly higher (*P* = 0.002) ratio with values of 0.538 ± 0.006 compared to 0.476 ± 0.006 in the spinach at T45, indicating a lower value of non-photochemical quenching in the former, perhaps consistent with the higher value of *A*. Returning to low light, the sugar beet *F*_v_′/*F*_m_′ values remain significantly higher than the spinach with values at T75 of 0.737 ± 0.002 compared to 0.708 ± 0.004.

Sugar beet had a greater average PSII operating efficiency (ΦPSII) in the light (*P* = 0.006) consistent with the higher values of *A* (Fig. 5B). ΦPSII was significantly greater (*P* = 0.042) at the end of the initial low light response (T7–T15) in the middle of the high light response (T26–T39, T42–T44) and consistently in the low light period (T46–T75) with a steady-state value at T75 of 0.708 ± 0.003 compared to 0.661 ± 0.006.

The level of photochemical quenching measured as *q*_p_ (Fig. 5C) was not significantly different when averaged over the entire response cycle. There was a trend (*P* = 0.062) of greater *q*_p_ in the sugar beet through all of the second low light period (T46–T75), with a steady-state value in this period of 0.960 ± 0.002 compared to 0.934 ± 0.004 in the spinach.

NPQ_t_ was higher (*P* < 0.001) in the spinach than sugar beet at 2.5 compared to 1.9, respectively, averaged over all time points T1–T75, driven by differences under the high light and subsequent low light period (*P* < 0.001). Under high light NPQ_t_ increased (*P* < 0.001) in both the sugar beet and the spinach and returned to levels comparable to pre the high light when the PPFD was decreased at T45 **[see****Supporting Information—Fig. S2A****]**.

### Stomatal anatomy

**Figure 6.**
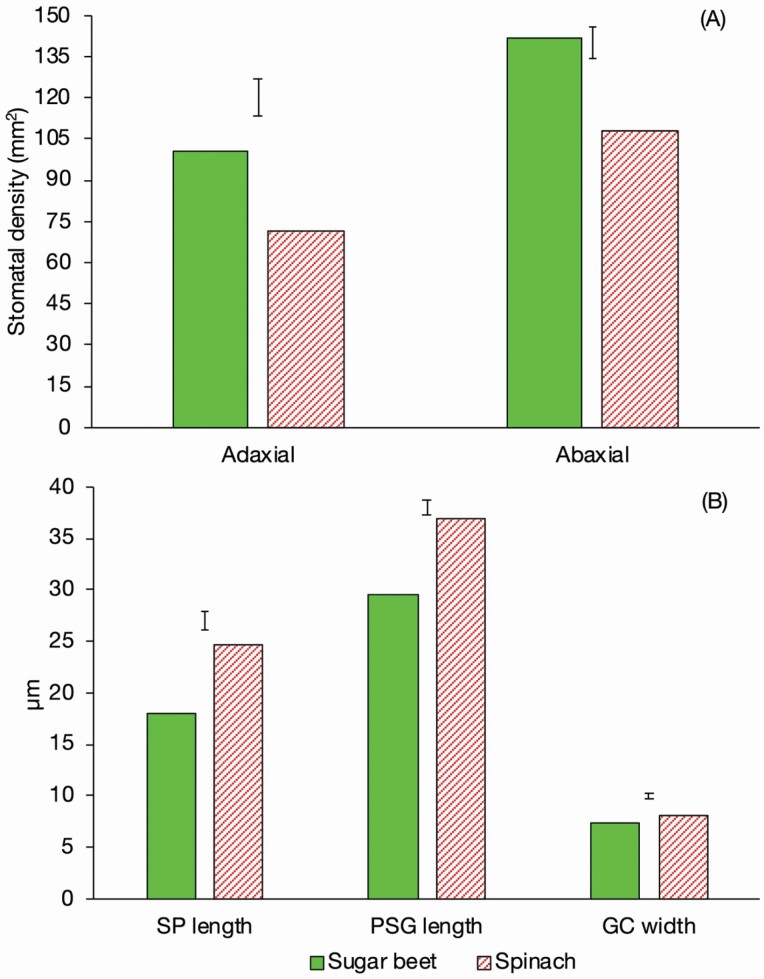
(A) The SD of the adaxial (*P* < 0.001, LSD = 6.90) and abaxial (*P* < 0.001, LSD = 5.90) leaf surface of spinach and sugar beet measured under optimal conditions. *n* = 8 sugar beet and 8 spinach. (B) The SP length (*P* < 0.001, LSD = 0.864), PSG length (*P* < 0.001, LSD = 0.761) and GC width (*P* = 0.003, LSD = 0.217) of sugar beet and spinach measured under optimal conditions. *n* = 8 sugar beet and 8 spinach.

Assessing SD and SS can provide an estimate of the maximum rate of *g*_s_ a plant can attain and, in this case, can be compared to the estimated values from the modelled LL.4 curves. The sugar beet had significantly greater SD (*P* < 0.001) than the spinach on both the adaxial and abaxial leaf surface (Fig. 6A). Sugar beet had a smaller SS than spinach with all three parameters measured being significantly less, SP length (*P* < 0.001), PSG length (*P* < 0.001) and GC width (*P* = 0.003) (Fig. 6B). These parameters were then used to calculate the theoretical maximum stomatal conductance of the adaxial and abaxial leaf surface using the model of Franks and Beerling (2009) which were combined to produce an overall average. The theoretical maximum to H_2_O was 2.87 mol m^−2^ s^−1^ and 2.84 mol m^−2^ s^−1^ and to CO_2_ which was 1.79 µmol m^−2^ s^−1^ and 1.78 µmol m^−2^ s^−1^ in beet and spinach, respectively. There was no significant difference between sugar beet and spinach in either parameter which supports the OE*g*_smax_ value calculated from the LL.4 curves.

### Light dynamic responses under drought

**Figure 7.**
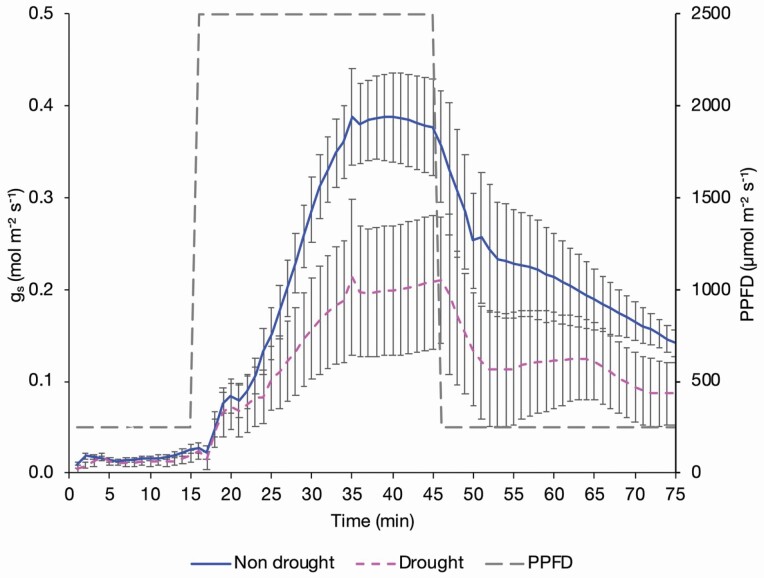
The stomatal conductance of non-drought and droughted sugar beet plants exposed to changing PPFD. Plants were exposed to a PPFD of 250 µmol m^−2^ s^−1^ for 15 min, 2500 µmol m^−2^ s^−1^ for 30 min and 250 µmol m^−2^ s^−1^ for 30 min, measured using an infrared gas analyser (LI-6800, LI-COR, Lincoln, NE, USA) with measurements logged every minute. These data were used to plot LL.4 curves and estimate stomatal speed. Error bars show SE ±, *n* = 4 non-droughted and 4 droughted sugar beet.

**Figure 8.**
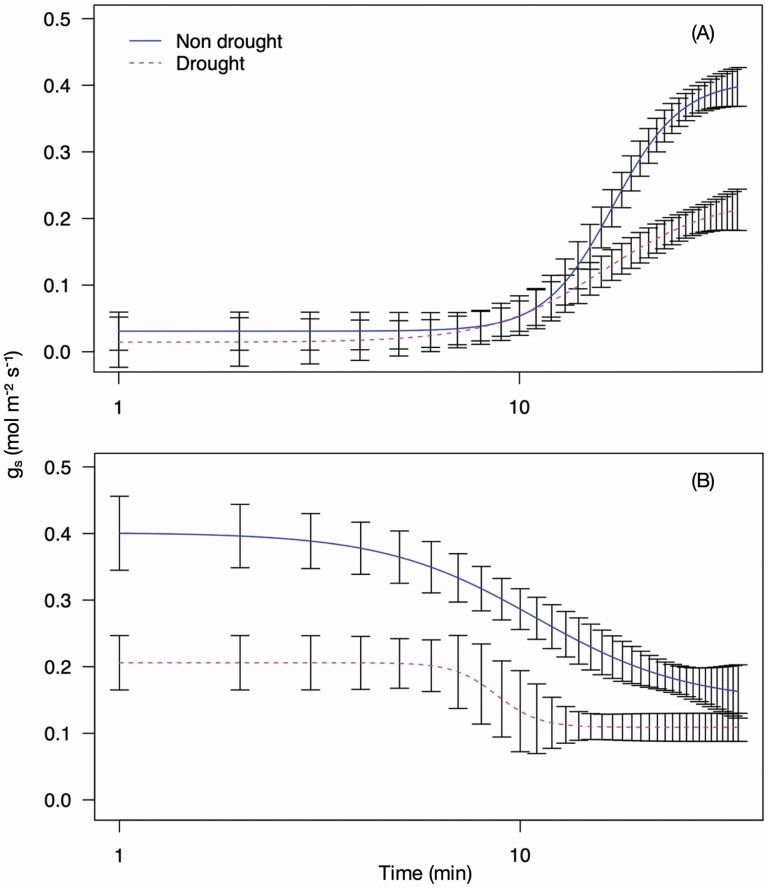
The LL.4 curves of stomatal conductance (*g*_s_) of non-drought and droughted sugar beet. Stomatal conductance was measured using an infrared gas analyser (LI-6800, LI-COR, Lincoln, NE, USA) and fitted using plotted using the DRC package (Ritz *et al.* 2015) in the statistical programming and graphics package R (R Core Team 2019). The plants were exposed to a PPFD of 250 µmol m^−2^ s^−1^ for 15 min, 2500 µmol m^−2^ s^−1^ for 30 min and 250 µmol m^−2^ s^−1^ for 30 min. Curve (A) shows the curve fitted when using the measurements taken during the last 5 min of the initial low light period and the 30-min high light period. Curve (B) shows the curve fitted when using the measurements taken during the last 5 min of the high light period and the 30 min low light period. The curves were identified as being significantly different (*P* < 0.001) using a two-way ANOVA. Error bars show SE ±, *n* = 4 non-droughted and 4 droughted sugar beet.

Sugar beet was selected for a focused analysis of dynamic responses to light under water deficit (drought) conditions. Droughted plants wilted and showed an altered stomatal response (Fig. 7). The fitted LL.4 curves of the non-droughted and droughted plants showed a significant difference (*P* < 0.001) in both stomatal opening (Fig. 8A) and closing phases (Fig. 8B). The droughted beet had a similar Og_s_50 to the non-droughted beet with an estimated time of 16.32 ± 2.47 min compared to 17.13 ± 0.71 min for the non-droughted, but with a slower rate of opening of 3.05 ± 1.39 compared to 5.11 ± 1.14 in the non-droughted (Table 2). This slower rate of response was associated with the reduced OE*g*_smax_ of the sugar beet of 0.23 ± 0.04 mol m^−2^ s^−1^ compared to 0.41 ± 0.02 mol m^−2^ s^−1^ for the non-droughted, which were close to the measured *g*_smax_ at T45 (Table 2). Returning to low light the droughted sugar beet reacted faster to close stomata with a Cg_s_50 of 8.73 ± 1.44 min and a rate of response of 8.12 ± 9.96 compared to 10.92 ± 2.57 min and 2.28 ± 1.17 for the non-droughted beet (Table 2). The OE*g*_smin_ and CE*g*_smin_ values were similar and close to the measured *g*_smin_ at T11 and T75, respectively (Table 2), highlighting that *g*_s_ values were not affected by water stress under low light, but were again estimated to be greater for the closing curve because the plants had acclimated to high light (Table 2).

**Table 2.** Estimated *g*_s_ parameters from LL.4 curves of non-droughted and droughted sugar beet exposed to stepwise changes in light to induce stomatal opening (250 to 2500 µmol m^−2^ s^−1^ PPFD) and closing (2500 to 250 µmol m^−2^ s^−1^ PPFD), with measured *g*_smin_ and *g*_smax_ values for comparison. The average LL.4 curves of non-droughted and droughted sugar beet, plotted from four replicates each, were analysed using two-way ANOVA and shown to be significantly different (*P* < 0.001).

		Non-drought			Drought		
Parameter	Units	Output	SE	*P*-value	Output	SE	*P*-value
Opening							
OE*g*_smin_	mol m^−2^ s^−1^	0.03	0.01	<0.001	0.01	0.02	ns
OE*g*_smax_	mol m^−2^ s^−1^	0.41	0.02	<0.001	0.23	0.04	<0.001
Og_s_50	min	17.13	0.71	<0.001	16.32	2.47	<0.001
Slope		5.11	1.14	<0.001	3.05	1.39	<0.001
T11 *g*_smin_^a^	mol m^−2^ s^−1^	0.02	0.00	–	0.01	0.00	–
T45 *g*_smax_^b^	mol m^−2^ s^−1^	0.38	0.05	–	0.21	0.07	–
Closing							
CE*g*_smin_	mol m^−2^ s^−1^	0.15	0.05	<0.001	0.11	0.01	<0.001
CE*g*_smax_	mol m^−2^ s^−1^	0.40	0.03	<0.001	0.21	0.02	<0.001
Cg_s_50	min	10.92	2.57	<0.001	8.73	1.44	<0.001
Slope		2.28	1.17	ns	8.12	9.96	ns
T75 *g*_smin_^c^	mol m^−2^ s^−1^	0.14	0.01	–	0.09	0.03	–

^a^Measured *g*_smin_ at T11 (pre-high light).

^b^Measured *g*_smax_ at T45 (during high light).

^c^Measured *g*_smin_ at T75 (post-high light).

### Assimilation and WUE_i_ in droughted sugar beet

**Figure 9.**
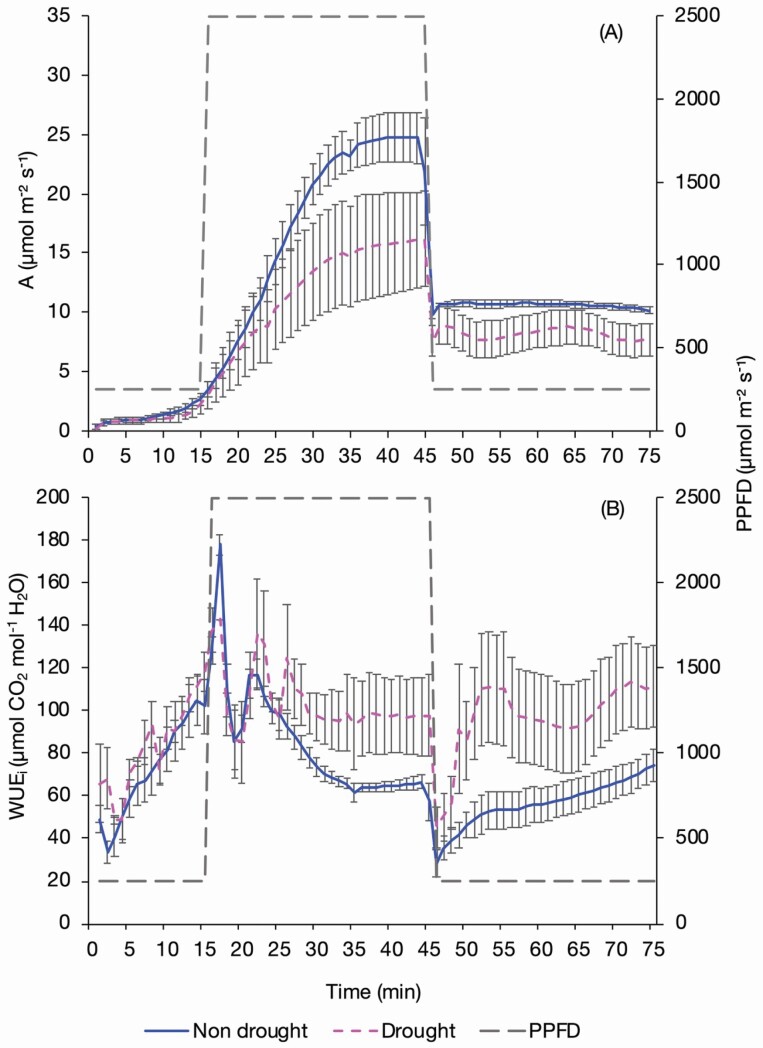
The assimilation (A) and WUE_i_ (B) of non-drought and droughted sugar beet plants exposed to changing PPFD. Plants were exposed to a PPFD of 250 µmol m^−2^ s^−1^ for 15 min, 2500 µmol m^−2^ s^−1^ for 30 min and 250 µmol m^−2^ s^−1^ for 30 min, measured using an infrared gas analyser (LI-6800, LI-COR, Lincoln, NE, USA) with measurements logged every minute. Error bars show SE ±, *n* = 4 non-droughted and 4 droughted sugar beet.

There was a trend of reduced *A* (*P* = 0.068) in droughted sugar beet under high light (T16–T45) and averaged over the entire response curve *g*_s_ was significantly lower (*P* = 0.023) in the droughted beet (Fig. 9A). This resulted in a lower average ratio of *g*_s_ to *A* and therefore a trend (*P* = 0.083) of higher WUE_i_ in the droughted beet compared to the non-droughted beet from T26 onwards, meaning that the decline in *g*_s_ was not proportional with the decline in *A* (Fig. 9B).

There was no significant difference in the performance of PSII in the droughted sugar beet despite water stress, with no significant differences in *F*_v_′/*F*_m_′, ΦPSII or *q*_p_ (*P* > 0.05) between the non-droughted and droughted sugar beet.

No significant differences in NPQ_t_ were evident between the non-droughted and droughted sugar beet but NPQ_t_ did significantly increase (*P* < 0.001) under high light and decease under the subsequent low light **[see****Supporting Information—Fig. S2B****]** as was evident as in the beet and spinach comparison.

### **The use of dose**–**response package to fit LL.4 curves to characterize stomatal opening**

The fitting of an LL.4 curve using the dose–response package provided a quantifiable comparison between the sugar beet and spinach responses and is similar to the approach of Drake *et al.* (2013). The stability of the control of VPD and air temperature at the low light and high light levels prevented VPD being a factor in the stomatal response and ensures that light alone was the driver of stomatal control in the plants studied. There are little published data on spinach and beet *g*_s_ but the OE*g*_smax_ of 0.45 mol m^−2^ s^−1^ is identical to the control values produced by Downton *et al.* (1985) when assessing spinach responses to salinity. Whilst the OE*g*_smax_ values for sugar beet are supported by the results of Katerji *et al.* (1997) who identified *g*_smax_ at 0.46 mol m^−2^ s^−1^ which is close to the 0.48 mol m^−2^ s^−1^ estimated here. The OE*g*_smin_ values are consistent with the values published for C_3_ plants by (Flexas *et al.* 2002) and for spinach by the observations of Delfine *et al.* (1998). The use of these values as the upper and lower limit to estimate the speed of stomatal responses both with regards to opening and closing is therefore justified.

## Discussion

### The response of sugar beet and spinach to changes in light intensity

Sugar beet had a high SD and small SS which may have contributed to fast stomatal responses to changes in light intensity, enabling *g*_smax_ and *A*_max_ to be reached more rapidly than in spinach, and a reduced disconnect between *g*_s_ and *A*. Therefore, the hypothesis that that sugar beet has slow stomatal responses attributed to a low SD and large SS is rejected.

As sugar beet stomata were faster to open in response to light compared to spinach, high levels of transpiration were quickly reached (Fig. 2). This coupled with the use of osmotic adjustment as Ψ _L_ falls (McCree and Richardson 1987), as opposed to stomatal closure, may contribute to making the plant highly susceptible to wilting. The key role that transpiration plays in sugar beet wilting is supported by the findings of Kohl and Cary (1969) who observed that high light drives wilting in sugar beet and that wilting severity can be reduced by constant mist irrigation throughout the day. The high rate of transpiration is also likely to be coupled with other traits which prevent adequate water uptake to maintain leaf turgor, such as mesophyll thickness and leaf vein arrangement (Sack and Holbrook 2006), which also supports the observations of wilting in the field, even when water is freely available. Therefore, it is not large, slow stomata leading to excessive water loss during stomatal closure but small, fast-opening stomata, with a greater magnitude of response under transient light than the spinach, which enables high rates of transpiration and photosynthesis and is likely to be a driver of wilting in sugar beet. Additionally, as VPD was kept stable at high light but *g*_s_ increased it is evident that light is a strong driver of stomatal responses in sugar beet, especially it is less responsive to reductions in Ψ _L_ due to its anisohydric behaviour. This may be relevant to sugar beet’s requirement for high rates of biomass production driven by high rates of photosynthesis. Under adequate water and high light, photosynthesis is often limited by the amount of photosynthetic components per unit leaf area, especially the enzyme Rubisco (Evans 1986). High stomatal conductance values are needed to drive these high assimilation rates, perhaps further increasing the likelihood of wilting.

The ability of sugar beet to reach *A*_max_ and *g*_smax_ faster than spinach alongside the increase in transpiration and the concurrent levels of high WUE_i_ suggest that, even though sugar beet wilts under high light levels, the plant is maximizing its use of the available resources (Mrad *et al.* 2019). Anisohydric woody species have previously been shown to have fast stomatal responses to light but at a cost of reduced WUE_i_ (Meinzer *et al.* 2017), but in this study the sugar beet WUE_i_ was comparable to the isohydric spinach despite faster stomatal responses as the balance between *g*_s_ and *A* was maintained and excessive *g*_s_ minimized. Plants that osmotically adjust have greater tolerance to water stress and this contributes to the ability of the plant to maintain photosynthetic performance, even when stomata remain open and Ψ _L_ falls (Ludlow 1987). In addition to this, a high rate of transpiration leads to evaporative cooling which initially protects the plant’s photosynthetic apparatus (Franks and Beerling 2009) before wilting. In comparison, the spinach is conserving water through a slower response but is not able to maximize the rate of *A*. Within the 30 min of high light intensity spinach only achieved 80 % of *g*_smax_, while sugar beet achieved 96 %. In the field, light intensity can constantly fluctuate due to the movement of clouds and the sun’s relative position throughout the day. The response of the beet may be optimal in these conditions as it is able to quickly open stomata to maximize *A* whilst closing rapidly to reduce the disconnect between *A* and *g*_s_ (Lawson and Weyers 1999; Lawson *et al.* 2010; McAusland *et al.* 2016; Vialet-Chabrand *et al.* 2017). Conversely, the spinach would not respond fast enough to maximize its use of the higher light intensity in rapidly changing light conditions. On a consistently bright day, however, the spinach’s more conservative response may be optimal to conserve water and reduce the likelihood of water stress throughout the day.

To ensure the anisohydric response and subsequent wilting is not detrimental to plant survival, sugar beet must maximize carbon fixation. The rise in *A* in response to an increase in PPFD, termed photosynthetic induction, is the summation of a combination of processes, including (but not limited to) the rate of Rubisco activation and stomatal opening (Kaiser *et al*. 2016). Rapid induction requires efficient photosynthesis to optimize light capture and maximize carbon fixation. Sugar beet demonstrated significantly higher maximum (*F*_v_′/*F*_m_′) and operating (ΦPSII) PSII efficiency when compared to spinach at both high and low PPFD. This is also evident in the higher values of *q*_p_ in sugar beet when recovering from the exposure to high light which demonstrates a greater proportion of open reaction centres in sugar beet, suggesting lower levels of NPQ investment (Murchie and Lawson 2013). Lower investment in NPQ means sugar beet is vulnerable to photoinhibition but avoids over protection of PSII, and is therefore capable of high photosynthesis rates and productivity (Kromdijk *et al.* 2016). This may be optimal for sugar beet as it is biennial, so needs to be highly productive for fast growth, and is adapted to latitudes away from the equator where PPFD is reduced and therefore photoinhibition rates are lower compared to latitudes closer to the equator.

There is a negative correlation between SS and SD across many species and conditions (Franks *et al.* 2009; Doheny-Adams *et al.* 2012) with more, smaller stomata enabling a greater rate of passage of CO_2_ into the mesophyll for assimilation as the length of the diffusion pathway is reduced (Franks and Farquhar 2007). However, in this study the *g*_smax_, from both the OE*g*_smax_ from the LL.4 model and the theoretical *g*_smax_ calculated from the stomatal anatomy is estimated to be only 0.03 mol m^−2^ s^−1^ higher in the sugar beet than the spinach. This suggests that it is a difference in the speed of stomatal opening, rather than the SD, that drives the difference observed between the beet and spinach in response to the changes in light intensity (Vialet-Chabrand *et al.* 2017). This is further supported by the sugar beet stomata being smaller, which enables them to react faster, as less ions and water movement is needed to drive changes in GC turgor (Hetherington and Woodward 2003; Drake *et al.* 2013). Additionally, when the SD and SS were used to calculate maximum conductance, using the Franks *et al.* (2009) model, there was no significant difference in the estimated maximum stomatal conductance between the sugar beet and spinach supporting the E*g*_smax_ and E*g*_smin_ from the LL.4 curves. The ability of spinach to reach a similar *g*_smax_ could be explained by the greater SS leading to a slower stomatal response but larger maximum stomatal aperture, but this relationship is not present in all species (Büssis *et al.* 2006; Doheny-Adams *et al.* 2012; Monda *et al.* 2016).

### The effect of water stress on the response of sugar beet to changes in light

Water stress altered the speed of stomatal response with slower opening and faster closing, compared to the well-watered plants which increased WUE_i_ at the expense of carbon fixation, as hypothesized. However, the magnitude of the stomatal response in the droughted sugar beet was greater than expected.

The reduction in *g*_smax_ in the droughted beet shows that the maximum stomatal opening, or the stomatal conductance under any given PPFD, is lower in water-stressed plants. The reduction in *g*_s_ also limits *A*_max_ as the rate of CO_2_ uptake is reduced as ribulose biphosphate synthesis can be inhibited (Tezara *et al.* 1999). The results of Ober *et al.* (2005) also show a reduction in the observed maximum assimilation rate *A*_max_ under drought across genotypes, with evidence of reductions greater than 50 %, whilst in this study the average reduction in *A*_max_ was 44 % under drought. The slower stomatal response under drought and relatively faster closing than opening has also been observed in French beans (*Phaseolus vulgaris*) and was driven by a greater sensitivity to plant Ψ _L_ (Barradas *et al.* 1994) which was not assessed in this experiment and the driver in sugar beet may be different due to anisohydry and the reduced sensitivity to Ψ _L,_ which could be explored further. As VPD was kept stable it is evident that water-stressed sugar beet reduce the magnitude of the stomatal response to changes in light compared to non-water-stressed beet. The observation that there was still a response from the droughted sugar beet to the high light shows that, even under severe water stress, where wilting was evident, the plant is still able to respond to environmental changes and effectively photosynthesize. The ability of the droughted plants to maintain a similar *F*_v_′/*F*_m_′, ΦPSII and *q*_p_ to the non-droughted also shows that in sugar beet wilting is not necessarily detrimental to PSII and therefore the photosynthetic apparatus of the plant. This may be linked to sugar beet’s anisohydric response, enabling photosynthesis to continue as Ψ _L_ declines. The reduction in *g*_s_ and *A* causes the ratio of the gradients for CO_2_ uptake and H_2_O loss to increase which also leads to increases in WUE_i_. Therefore, reducing stomatal aperture will lead to increases in WUE_i_ which are beneficial under drought to make the most of any available water, and have been previously reported in sugar beet (Rytter 2005; Bloch *et al.* 2006;Topak *et al.* 2011).

In the UK, intermittent rather than terminal drought is common (Jaggard *et al.* 1998). The ability of sugar beet to respond to light, even when drought-stressed, is therefore beneficial as further water stress due to transpiration, as the stomata open for CO_2_ uptake, is less risky in an intermittent drought than a terminal drought. The fact that drought stress is rarely terminal in the UK also suggests that the wilting response previously mentioned is not necessarily detrimental to the crop, as the temperate climate will enable rapid recovery, whilst the plant has maximized its use of the available light for carbon gain.

### Can we optimize the stomatal response of sugar beet?

Both the rapid response of sugar beet to high light and its ability to respond to light even when severely drought-stressed may be attributed to its ancestry. Sugar beet is descended from sea beet which is found across Europe. A study by Ribeiro *et al.* (2016), demonstrated the ability of some sea beet plants, found in differing environments in Portugal, to rapidly recover from severe drought and salinity stress. The greater level of allelic diversity in the sea beet suggests that the rapid response of commercial sugar beet, as shown in this study, could be changed through introgressing traits from the more conservative wild types. In addition to this, differences in drought tolerance and associated traits are evident, even within the current commercial sugar beet varieties (Ober *et al*. 2004, 2005; Luković *et al.* 2009; Rajabi *et al.* 2009; Schickling *et al.* 2010) and may provide another avenue to identify plants which have different levels of stomatal control. As discussed earlier, a more conservative sugar beet may be more productive in water-limited conditions, such as dry years in the UK where losses of up to 25 % (Jaggard *et al.* 1998) are evident and other areas of cultivation in Europe (Jones *et al.* 2003) and the USA (Cooley *et al.* 2015).

## Conclusions

Sugar beet responded more rapidly to increased light than spinach, likely due to smaller stomata. However, the lower SD and greater SS was not a limitation to the OE*g*_smax_ of the spinach. The ability of sugar beet to react quickly compared to spinach enables *A*_max_ and *g*_smax_ to be reached rapidly but this may result in high levels of water loss through transpiration which, coupled with the anisohydric response, could drive wilting. Although this response may not be optimal when the weather is consistently dry, as soil water is used up rapidly, terminal drought is not usually of concern in most countries that cultivate sugar beet. The ability of sugar beet to maintain a low level of *A*, even when drought-stressed and without damage to the photosystems, also highlights its suitability to the short-term drought events common in many areas of cultivation. As the climate changes, and prolonged dry periods become more frequent, it may be necessary to utilize sea beet traits to breed more water conservative commercial sugar beet varieties.

## Supporting Information

The following additional information is available in the online version of this article—

**Figure S1.** The VPD of non-drought and droughted sugar beet plants (A) and droughted and non-droughted sugar beet (B) exposed to changing PAR of 250 μmol m^−²^ s^−1^ for 15min, 2500 μmol m^−²^ s^−1^ for 30min and 250 μmol m^−²^ s^−1^ for 30min, with measurements logged every minute and measured using an infrared gas analyser (Li6800, LI-COR, Lincoln, Nebaska, USA). (A) n = 8 Sugar beet and 8 spinach, (B) n = 4 non-droughted and 4 droughted sugar beet. Error bars show standard error.

**Figure S2.** The NPQt of non-drought and droughted sugar beet plants (a) and droughted and non-droughted sugar beet (b) exposed to changing PAR of 250 μmol m^−²^ s^−1^ for 15min, 2500 μmol m^−²^ s^−1^ for 30min and 250 μmol m^−²^ s^−1^ for 30min, with measurements logged every minute measured using an infrared gas analyser (Li6800, LI-COR, Lincoln, Nebaska, USA). Error bars show SE±, (a) n = 8 Sugar beet and 8 spinach, (b) n = 4 non-droughted and 4 droughted sugar beet.

plaa067_suppl_Supplementary_Figure_S1

plaa067_suppl_Supplementary_Figure_S2

plaa067_suppl_Supplementary_Data

## Data Availability

The raw data are available as Supporting Information.

## Notes

**Form & Function. Chief Editor:** Kate McCulloh
