# Supplementary material for: Anisohydric sugar beet rapidly responds to light to optimize leaf water use efficiency utilizing numerous small stomata
Source: AoB Plants. 2020 Dec 2;13(1):plaa067. doi: 10.1093/aobpla/plaa067 (PMC7780706; doi:10.1093/aobpla/plaa067)
Supplement: plaa067_suppl_Supplementary_Figure_S1 [file plaa067_suppl_supplementary_figure_s1.pdf]

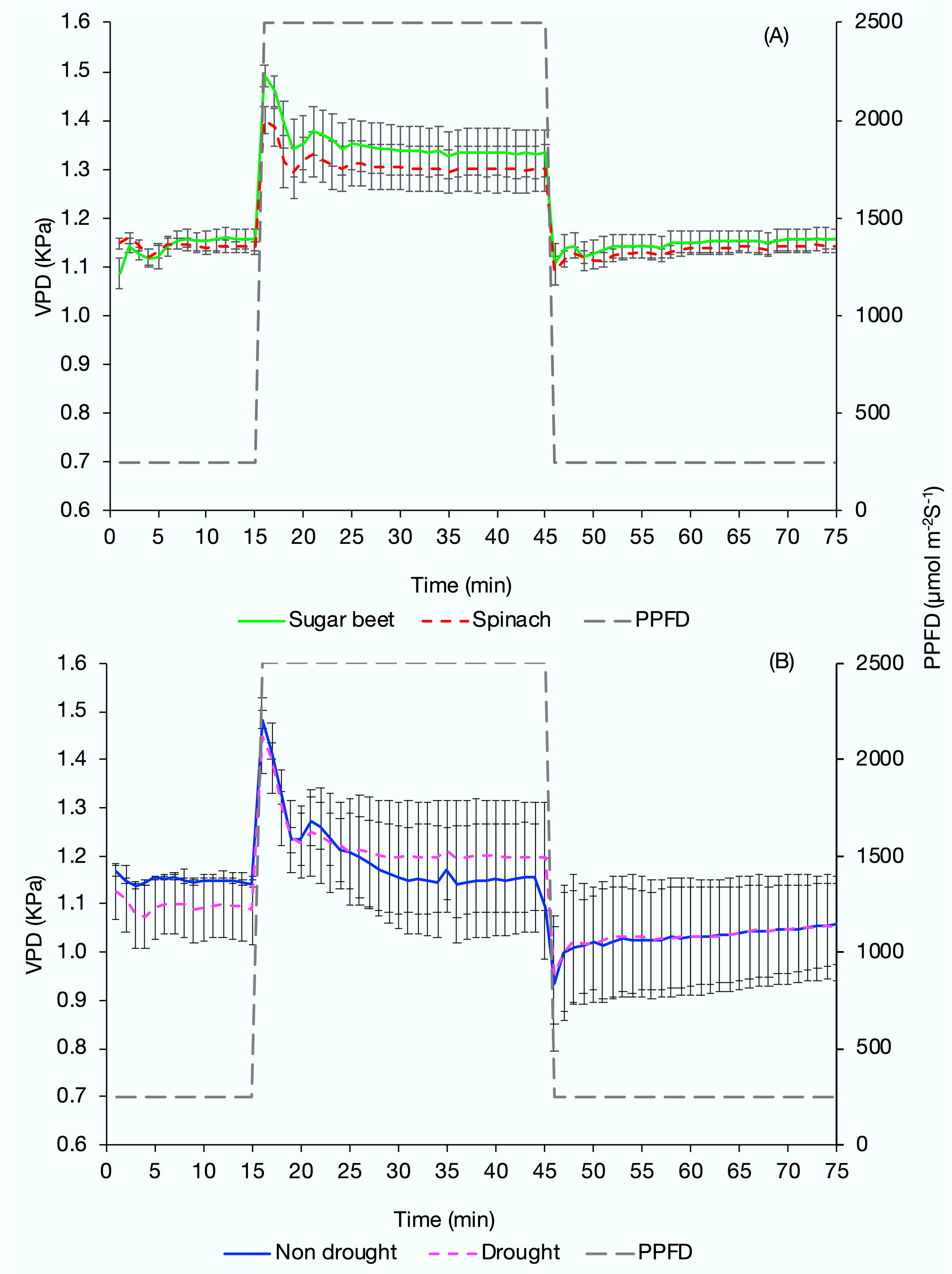

Figure S1. The VPD of non-drought and droughted sugar beet plants (A) and droughted and non-droughted sugar beet (B) exposed to changing PAR of 250 µmol m^{-2} s^{-1} for 15min, 2500 µmol m^{-2} s^{-1} for 30min and 250 µmol m^{-2} s^{-1} for 30min, with measurements logged every minute and measured using an infrared gas analyser (Li6800, LI-COR, Lincoln, Nebaska, USA). (A) n= 8 Sugar beet and 8 spinach, (B) n= 4 non-droughted and 4 droughted sugar beet. Error bars show standard error.
