# Supplementary material for: Anisohydric sugar beet rapidly responds to light to optimize leaf water use efficiency utilizing numerous small stomata
Source: AoB Plants. 2020 Dec 2;13(1):plaa067. doi: 10.1093/aobpla/plaa067 (PMC7780706; doi:10.1093/aobpla/plaa067)
Supplement: plaa067_suppl_Supplementary_Figure_S2 [file plaa067_suppl_supplementary_figure_s2.pdf]

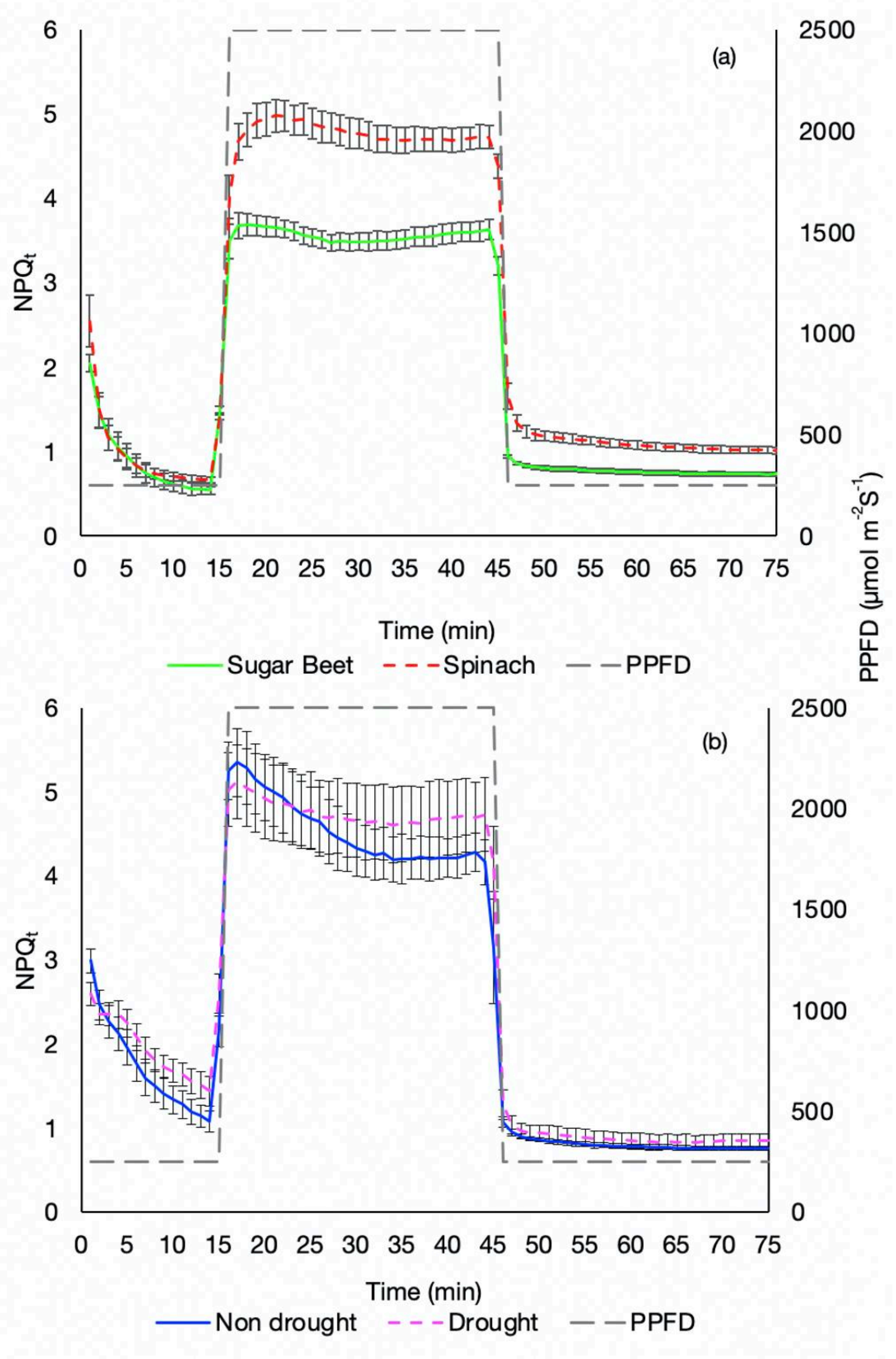


**Figure S2.** The NPQt of non-drought and droughted sugar beet plants (a) and droughted and non-droughted sugar beet (b) exposed to changing PAR of 250 µmol $m^{-2}$ $s^{-1}$ for 15min, 2500 µmol $m^{-2}$ $s^{-1}$ for 30min and 250 µmol $m^{-2}$ $s^{-1}$ for 30min, with measurements logged every minute measured using an infrared gas analyser (Li6800, LI-COR, Lincoln, Nebaska, USA). Error bars show SE±, (a) n= 8 Sugar beet and 8 spinach, (b) n= 4 non-droughted and 4 droughted sugar beet.
